# Honey bee-collected pollen is a potential source of *Ascosphaera apis* infection in managed bumble bees

**DOI:** 10.1038/s41598-019-40804-2

**Published:** 2019-03-12

**Authors:** Kleber de Sousa Pereira, Ivan Meeus, Guy Smagghe

**Affiliations:** Ghent University, Faculty of Bioscience Engineering, Department of Plants and Crops, Lab of Agrozoology, Coupure Links 653, Ghent, B-9000 Belgium

## Abstract

The trade of bumble bees started in the early nineties for pollinator-dependent greenhouse plants. Nowadays, its rearing and transport have received public attention, since managed bees can transfer pathogens to wild bee populations. Therefore, guaranteeing pathogen-free bumble bees is fundamental. The major protein source used in rearing facilities is honey bee-collected pollen. This can carry pathogens, however to date, solid data on the risk of this food source to the health of bumble bees is lacking. Here we performed a large pathogen screening of non-irradiated honey bee-collected pollen to discover particles infective to *Bombus terrestris*. We identified seven parasites (*Apicystis bombi*, *Ascosphaera apis*, *Crithidia mellificae, Nosema ceranae, Paenibacillus larvae* and two parasites resembling *Nosema thomsoni* and *Microsporidium* sp. *Oise*) and four viruses (CBPV, DWV, IAPV and SBV) in 17 pollen batches from two major European pollen source regions (Spain and Romania). *Ascosphaera apis* was capable of infecting bumble bees; the larvae showed similar symptoms to chalkbrood disease reported in honey bees. Bumble bee breeding facilities need to be cautious about the potential presence of this disease, which was originally reported in honey bees. Thorough diagnostic and control methods are needed, as risk of spillover to wild bee species is possible.

## Introduction

Bee domestication began with the honey bee, attractive for its production capacities of honey, wax and propolis. Nowadays, various bee species are managed, and their pollination services are exploited to form a ‘new’ pollination industry^1^. A good example is bumble bee management and trade, which started in the early nineties within enclosed facilities. The bumble bee market was initially focused on providing pollination services in greenhouses and has expanded to open-field pollination of bumble bee-visited crops^2^. Currently, the international bumble bee market is still expanding^2,3^, with pollination activities in more than sixty countries.

The health status of managed bumble bees is crucial in international trade. First, several viral and non-viral pathogens can reduce fitness, resulting in shorter life span, reduced learning ability^4–6^ and likely reduced pollination efficiency^5^. Second, commercial bees may act as a reservoir of pathogens and can be responsible for the transmission of pathogens from commercial bees to wild pollinators^7–9^. Such transmission is a specific case of pathogen spillover, more generally defined as a transmission driven by a species acting as a pathogen reservoir. Bee transport can facilitate the presence of such reservoirs influencing natural host-parasite dynamics, and therefore influence host populations within natural ecosystems^10^. With regard to spillover, it has been argued that it can have devastating effects on endemic bee fauna^7,11^. Furthermore, other stressors harm bee populations (e.g. land-use and pesticide), which can act in synergy with parasite stress^12^. For example, *Nosema bombi* prevalence in declining bumble bees (*Bombus occidentalis, B. pensylvanicus, B. affinis* and *B. terricola*) in North America is correlated with fungicide presence^12^.

Pathogen-free bumble bee management is essential in order to limit spillover dynamics or introductions of new parasitic species or genetic strains. Although the rearing of bumble bees occurs indoors with no direct contact with the outdoor environment, there are still two main routes of pathogen influx into the breeding facilities. The first potential transmission route is the bumble bee itself, as a certain genetic stock is needed to reach the desired production capacities. Queens first undergo quarantine measures and new offspring can be tested to obtain pathogen-free nests and daughter queens. Thus, pathogen-free facilities are a matter of thorough diagnostics and time/cost investment efforts. A second potential influx route is the honey bee-collected pollen (pollen pellets on the corbicula of honey bees)^5,13,14^, which is used as the main protein source of the bumble bee’s diet. A recent study on two pollen batches showed that honey bee-collected pollen was a probable source of infective *Apicystis bombi* oocysts^5^. This is surprising since *A. bombi* was mainly known as a bumble bee parasite. Then again, very little is known about the natural host range of many bee parasites^14^ and the occurrence in honey bees had been reported sporadically^15^. These results led us to speculate that honey bee-collected pollen could serve as potential source for other parasites and viruses. Indeed, many pathogens originally reported in honey bees were later also found in bumble bees in Europe and North America^4,6,16–19^.

Sanitary control measures on honey bee-collected pollen are an important aspect of good bumble bee rearing practices. Yet no thorough prevalence study of parasites and viruses in honey bee-collected pollen for commercial purpose has been performed to date. The aim of this study was to perform screening for wide variety of parasites and viruses on pollen used for bumble bee rearing (N = 17 different pollen batches). The use of broad-range PCR diagnostics, where possible, allowed us to screen for potential undiscovered threats. Finally, we performed a realistic oral infection test on a subset of parasite contaminated pollen batches to investigate the infectivity risk.

## Results

### Pathogen diversity in honey bee-collected pollen

**Figure 1:**
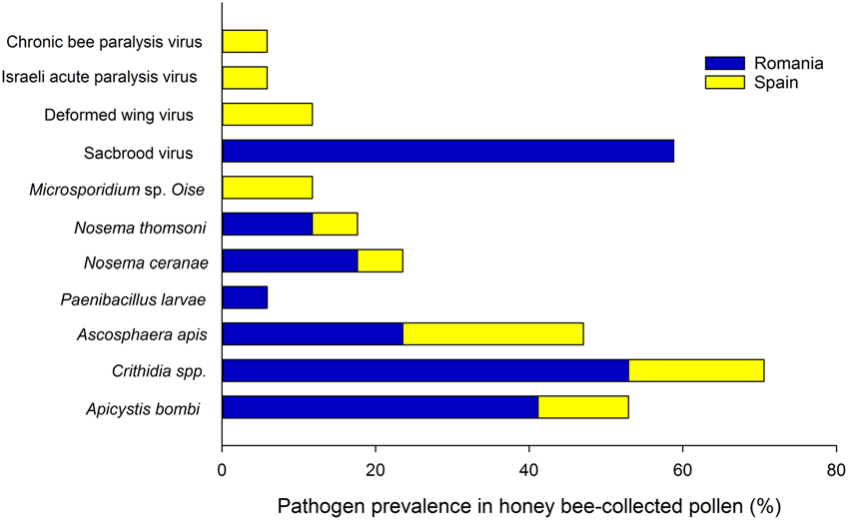
The cumulative prevalence in percentage of all the parasites retrieved in 17 pollen batches from Spain and Romania. Bars in yellow indicate pathogens identified in samples from Spain and in blue, the ones from Romania.

We screened a total of 17 different pollen batches for the presence of parasites and viruses; the batches originated from 4 different suppliers located in Romania and Spain. In total, 7 parasites and 4 viruses were detected (see Fig. 1). The most prevalent pathogen within all honeybee-collected pollen samples was *Crithidia* spp. (70.6%), Sacbrood virus (SBV) (58.8%), *A. bombi* (52.9%) and *Ascosphaera apis* (47.1%) followed by the Microsporidia *Nosema ceranae* (23.5%), *Nosema thomsoni* (17.6%), *Microsporidium* sp. *Oise* (11.7%) and the viruses Deformed wing virus (DWV) (11.7%) and Israeli acute paralysis virus (IAPV) and Chronic bee paralysis virus (CBPV) with 5.8%. Sequences of *N. ceranae*, *N. thomsoni* and *Microsporidium* sp. *Oise* had 99% identity with sequences from GenBank accession numbers AB860144.1, EU219086.1 and HM566197.1, respectively. All the parasites were retrieved in both regions (Romania and Spain); while *Paenibacillus larvae* was found only in one pollen batch from Romania. Furthermore, a minimum of 1 parasite and a maximum of 4 parasites was found per pollen batch tested. Concerning the viruses, SBV was highly present in the batches from Romania and this exclusively, while CBPV, IAPV and DWV were found only in suppliers from Spain (Fig. 1). *Nosema apis, N. bombi* and the viruses Kashmir bee virus (KBV), Acute bee paralysis virus (ABPV) and Black queen cell virus (BQCV) were not detected in any pollen sample.

### Infection risk of pollen to larvae and adults of bumble bee

In total we tested 7 different parasites *A. bombi, A. apis*, *Crithidia* spp., *N. ceranae*, *N. thomsoni*, *Microsporidium* sp. *Oise* and *P. larvae* in the infection assay. We only detected an active infection for *A. apis*. The known honey bee parasite *A. apis* was particularly prominent in the gut of bumble bee larvae. It was detected in three out of the four microcolonies that were supplied with pollen containing the parasite (Table 1). The other parasites *A. bombi*, *Crithidia* spp.*, N. ceranae*, *N. thomsoni*, *Microsporidium* sp. *Oise* and *P. larvae* were not detected in any bumble bee tissue, although they were present in the pollen.

**Table 1:** Number of infected living bumble bee larvae with *Ascosphaera apis* after feeding on contaminated pollen with this parasite (4 microcolonies) and one control microcolony with Irradiated-pollen.

Larva type inspected	N° of micro-colonies (n = 5)				
	with contaminated pollen				with irradiated pollen
	1	2	3	4	5
Infection analysis on living larvae (n = 3)	**+++**	**+++**	**+++**	**−**	**−**
Presence of dead larvae with symptoms	**−**	**+**	**+**	**+**	**−**

The symbol “+” indicates the number of infected gut tissues (N_max_ = 3) per pollen treatments and the symbol “−” the absence of infections (N = 3 larvae inspected). Afterwards, dead bee larvae were screened if chalkbrood manifested. Despite one contaminated colony (4) did not present infected living larva, it contained a dead larva with chalkbrood disease symptom.

### Infectivity of *A. apis* in bumble bee larvae

#### Microscopic identification and morphological description

**Figure 2:**
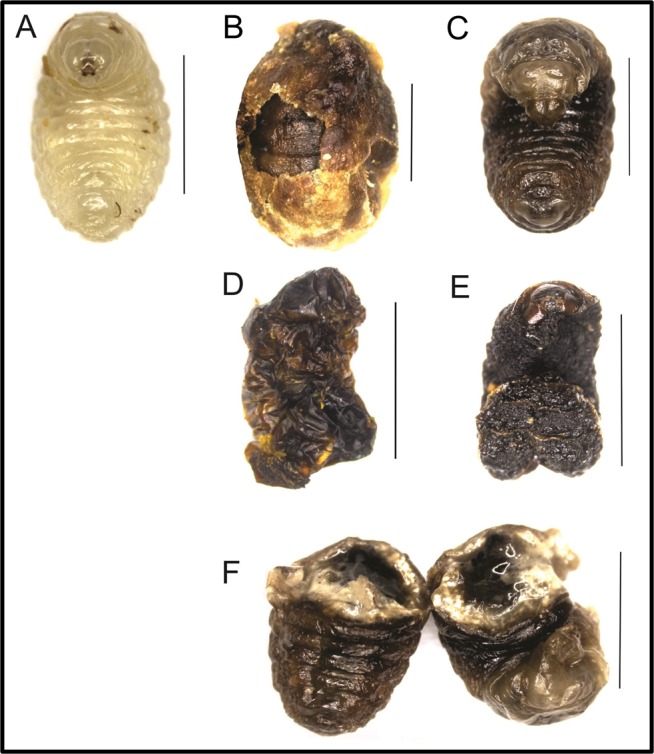
Different health conditions of *Bombus terrestris* larvae at 4^th^ instar. (**A**) Healthy bumble bee larva. (**B**–**F**) Bumble bee larvae showing symptoms of chalkbrood disease after ingestion of honey bee-collected pollen with *Ascosphaera apis*. (**B**) Unremoved larva contaminated with *A. apis* in closed cocoon by co-worker in colony. (**F**) A close up of an opened creamy larva infected with *A. apis*. Scale bars = 50 mm.

**Figure 3:**
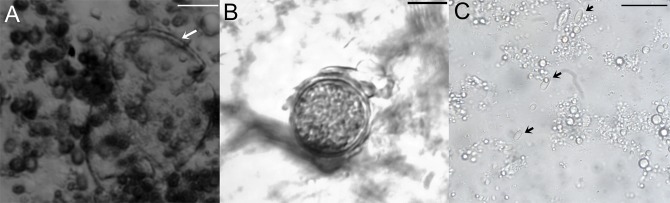
Stages of *Ascosphaera apis* infecting bumble bee larvae. (**A**) Close-up of an opened spore cyst. (**B**) A spore ball when released from a cleistotheca. (**C**) Bacilliform ascospores. Scale bars = 10 µm.

We detected larvae presenting symptoms still inside the cocoon in three out of the four microcolonies supplied with pollen with *A. apis*. One of these dead larvae was found in one contaminated colony that was negative for the infection test with living larvae. Two larvae were blackened and mummified showing common symptoms of chalkbrood disease as described within the host *Apis*^20,21^(Fig. 2) and one infected exhibiting an unusual symptom, distinctly creamy in appearance (Fig. 2C,F). In addition, we detected vegetative and reproductive stages of *Ascosphaera* fungi in these larvae (Figs 2 and 3). The measurements of the fungus were equivalent to this species^22–24^ presenting spore cysts with a diameter of 39.3–52.0 μm (average = 44.9 ± 3.8 μm), a spore ball with 12 μm in diameter and ascospores with lengths of 1.9–3.4 μm (average = 2.6 ± 0.3 μm).

#### Phylogenetic analysis

**Figure 4:**
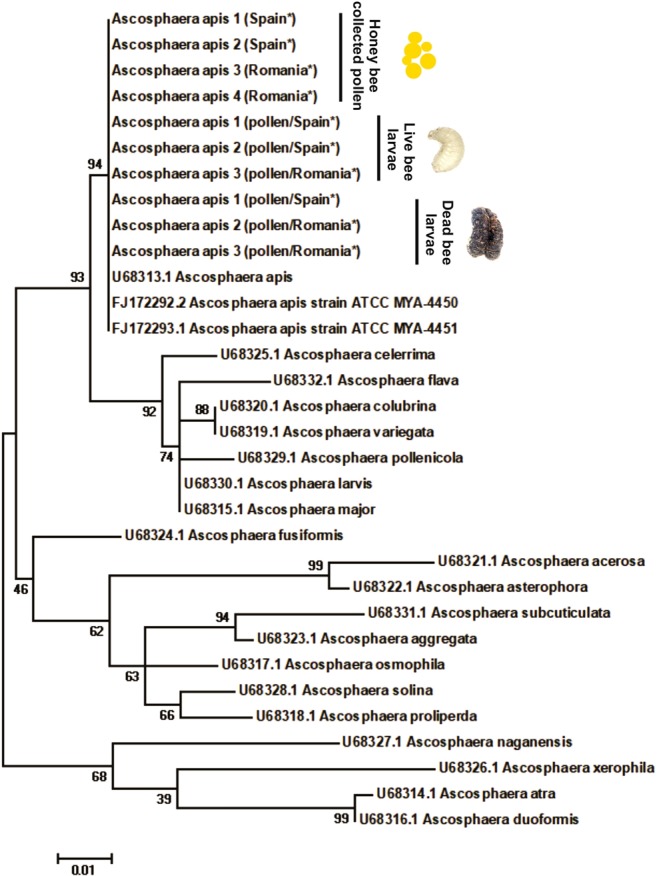
Phylogeny of the internal transcribed spacer region for selected *Ascosphaera* species. ITS sequences from this study were concatenated to strains of *A. apis* (ATCC MYA-4450, Genbank accession #FJ172292; ATCC MYA-4451, Genbank accession #FJ172293) together to another ITS from the genus *Ascosphaera*, Genbank accession #U68313.1 (see Anderson *et al*.^22^). Phylogenetic analysis performed using the maximum likelihood algorithm. Analyses was executed using 1000 bootstrap replicates.

All ITS sequences of *Ascosphaera* fungi found in honey bee-collected pollen, alive larvae, and symptomatic dead larvae matched 100% identity (336–345 bp fragments length) with ITS sequences from voucher strains of *A. apis* from Genbank database (NCBI). The phylogenetic analysis placed all sequences of *A. apis* found in pollen and larvae from this study together with existed database sequences (Fig. 4).

#### *Ascosphaera apis* quantification

**Figure 5:**
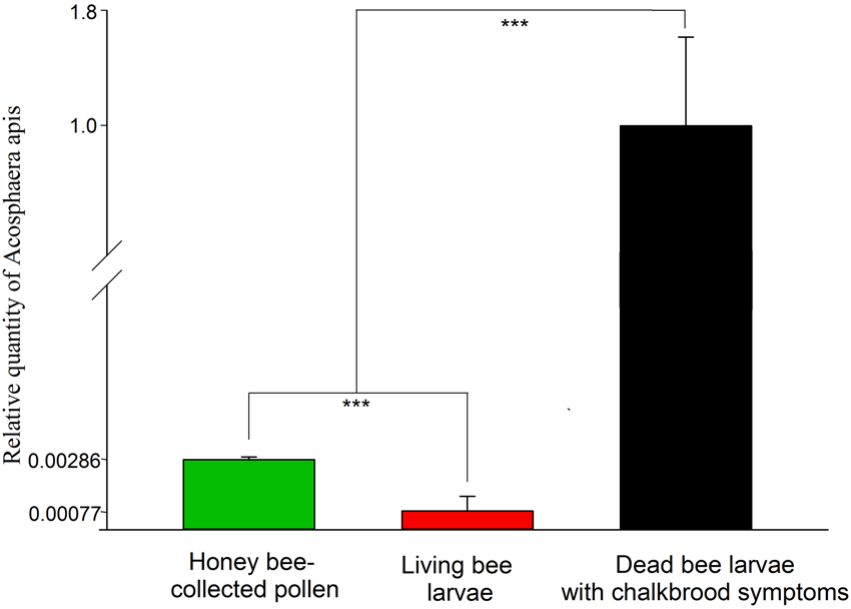
Specific qPCR measurement of 18S rRNA gene from *Ascosphaera apis*. Bars indicate the relative quantity (normalized to 1) of *Ascosphaera apis* transcripts in honey bee-collected pollen, alive larvae and symptomatic dead larvae. Error bars denote ± s.e.m. from six (n = 6), nine (n = 9) and three (n = 3) independent samples, respectively. Error flags are SE and the size are in accordance to the different scales of the y-axis. Statistical significance calculated using the independent samples *t*-test, ***P-value < 0.001.

The load of *A. apis* was significantly different among all the sample groups (honey bee-collected pollen, living larvae and dead larvae manifesting symptoms). The number of genome copies of the fungus was higher in dead larvae exhibiting symptoms compared to living larvae and pollen samples (*P* < 0.0001, Fig. 5).

## Discussion

The presence of pathogens in bumble bee breeding facilities receives attention mainly because of the potential disease transmission risk towards wild bees^3,5,8,14,25^. Although a main aspect, only a few studies looked at the pathogen influx routes into these sites. Honey bee-collected pollen can be identified as a potential introduction route of pathogens. However, to date, little knowledge is present to understand how substantial this problem is. This understanding is not only important in terms of regulation, for instance on the use of unsterilized pollen and its origin, but also provides valuable information for the sector in terms of sanitary controls within the facility. In this study, we found that each of the 17 pollen batches contained at least one parasite or virus (at max. 5). This highlights a widespread potential problem for bee breeding facilities. It is essential to assess the impact of each of these pathogens on bumble bees and more importantly assess the impact of pathogens able to infect multiple hosts (e.g. honey bees and bumble bees).

The clearest result is the infection of *A. apis* in the larvae of *Bombus terrestris*. ITS sequencing revealed that the *Ascosphaera* fungi detected in honey bee-collected pollen and in infected bumble bee larvae were identical with each other and with the *A. apis* sequences (#FJ172292, #FJ172293 and #U68313) deposited at GenBank. We did not only identify the presence of this parasite, but we could also demonstrate infection. We detected chalkbrood-like symptoms in *A. apis* positive larvae, which also contained higher parasite load. The massive growth of *A. apis* and the occurrence of symptoms seems to be causal, but it can also be triggered by co-occurring parasites. Since we only have three observations of symptoms, we cannot perform co-occurrence analysis and infer such exact causality. These results are important since the use of many reared bumble bee species in biological pollination worldwide poses a risk to wild pollinators if parasites are shared. For this reason, it is important to know the host preference and virulence of *A. apis*. To date, the established infection based on symptoms, fungus sporulation or via microscopy of this fungus, refers to larvae of the European honey bee, *Apis mellifera*^26^, the Asian honey bee, *Apis cerana*^27^, the carpenter bee *Xylocopa californica arizonensis* Cresson^28^ and *Xylocopa augusti*^21^. There is no definitive evidence that *A. apis* can infect wild bumble bee larvae, and anecdotal reports of the parasite are mainly associated with the rearing of bumble bees in artificial conditions. For instance, *Ascosphaera* spp. (no species identification) was isolated from a dead bumble bee larva reared in the laboratory^29^, where the authors speculated that honey bee-collected pollen could be the infection source. Recently, isolates of *A. apis* were detected in wild North American bumble bee queens of *Bombus griseocollis, B. nevadensis* and *B. vosnesenskii*^24^, again after bringing them into the lab, and thus there was no identification if the captured queens already had the infection or were infected by feeding on contaminated pollen. PCR diagnostics have identified *Ascosphaera* in many bee species, and so it was concluded that the fungus was very common in the environment of pollinators, yet mainly in the context of being vectored by non-host pollinators^17^. Here, we show a true infection in managed *B. terrestris*. We noticed sign of chalkbrood in *A. apis*-infected bumble bees which is a common symptom that was originally reported in the larvae of honey bees^30^. Bumble bee breeders should be aware of this fact, and chalkbrood symptomology and the presence of this parasite must be verified through implemented sanitary measures. It is important to highlight that the diseased larvae were not thrown out of their brood cell, and thus they were only spotted after opening the brood cell manually. Therefore, the eradication of this parasite from breeding facilities is important to prevent spillover toward wild bumble bees, as the managed bees could act as a parasite reservoir for *A. apis*.

We identified another pathogen namely *P. larvae*, that is typically associated with honey bees, but that is not known to infect bumble bees. This pathogen might represent a threat since it is likely to cause infection in breeding facilities. Thus, its presence in pollen samples is still alarming, and we consider the introduction of *P. larvae* as a potential danger. This bacterium is the etiological agent of the American Foulbrood (AFB), the most important disease of honey bees worldwide^31^. Due to its high virulence, honey bee hives need to be eradicated when diagnosed with this quarantine organism. We only had one positive sample to test potential transmission towards commercial bees through infection experiment and they remained negative. To the best of our knowledge, no wild bumble bees have been identified as harboring *P. larvae*. Yet, artificial infection with bacterial cultures is needed to perform a meaningful risk evaluation. We underline the importance of these common honey bee pathogens in the pollen for further specific study, as the breeding process of bumble bees with honey bee-collected pollen facilitates an artificial contact with a potential new host. The rearing process could facilitate spillover across species, which would be less likely to occur in natural conditions. Although possible, this latter point remains speculative.

The detection of microsporidium sequences related to *Microsporidium* sp. *Oise* and particularly *N. thomsoni* also presents an interesting finding. *Nosema thomsoni* is associated with moths^32,33^ and the ladybird *Harmonia axyridis*, and the parasite association with the latter is thought to threaten native ladybird species in Europe^34^. The 18S sequencing reveled matches (99%) with *N. thomsoni* from moths, with sequences within the *N. thomsoni* clade of *Nosema* species found in Asian bumble bees^35^, and with the sequence found in the solitary bee *Andrena vaga* in Belgium^36^ and recently in *Andrena haemorrhoa*^37^. We did not observe infectivity of any microsporidium or with *N. ceranae*-contaminated pollen. This could be attributed to a number of reasons: (1) the infectious potential can be lost due to the freezing process that the pollen undergoes for instance *N. ceranae* is known to be sensitive to cold temperature^38^, (2) the inoculation loads were not high enough, and/or (3) these parasites are not able to infect bumble bees. *Nosema thomsoni* can be regarded as a multihost parasite, and its presence in honey bee-collected pollen is more likely an environmental contamination of other pollinators visiting the flower before the pollen was collected by the honey bee. In this case, similar contact with this parasite also occurs in nature. Furthermore, the recent discovery of different *Nosema* species in bumble bees with use of molecular diagnostics cannot always be linked with true infections, as remarked by Brown^39^.

Based on the results, it is clear how problematic the influx of non-sterilized honey bee-collected pollen into facilities is, as the pollen from every supplier was contaminated with at least 3 pathogens. Although, many parasites of bees and insects found here did not establish infection, it does not suggest an invulnerability for those parasites. This first documentation of *A. apis* in *B. terrestris* larvae via feeding pollen is alarming and endorses the need of measures to reduce or prevent the entry of pathogens in commercial establishments. Pollen trade and transportation are therefore also important to be considered in the context of potential parasite spillover from commercial to wild bumble bees. We are aware that some bumble bee breeders advertise the use of gamma-irradiated pollen, which is a good practice. Nonetheless, to our knowledge, no legislation is existing yet on the sterility and sanitary control measures to import pollen or use it for insect breeding in general. We encourage further studies on sterilization measures and their efficacy for bee pathogens and potential trade-offs towards nutritional value.

## Materials and Methods

### Prevalence screening and diagnostics

#### Honey bee-collected pollen and Nucleic acids extraction

We assessed pollen intended to feed bumble bees in breeding facilities in Europe. In total 17 frozen pollen batches from four different suppliers in Spain and Romania were tested for viral and non-viral pathogens of bees. Sampling was carried out and obtained from May until July of 2016. Twenty pollen pellets from each pollen were pooled (approximately 0.3 g) in a 2 ml-microcentrifuge tube (Eppendorf). We added 800 µl of RLT buffer supplemented with β-mercapto ethanol (100/1; v/v) (RNeasy Mini Kit; Qiagen, Venlo, the Netherlands). After, the samples were homogenized for 2 min at 300 rpm and for 2 min at 200 rpm with 0.1 mm-diameter zirconia beads and two 5 mm diameter-metal beads in a Qiagen TissueLyser. The mixture was centrifuged for 2 min at 20000 g. For DNA extraction 200 µl of the supernatant was mixed with 400 µl of Lysis buffer G and 40 µl of Proteinase K, and incubated for 1 h at 52 °C with shaking (400 rpm). Further extraction was done according to the manufacturer’s protocol (Invisorb Spin Tissue Mini Kit, Protocol 1; Stratec, Berlin, Germany), DNA was stored at −20 °C until further use. RNA extractions were done starting with 200 µl of supernatant and 200 µl of 70% ethanol was added. Further extraction was done according to the manufacturer’s protocol (RNeasy Mini Kit; Qiagen), RNA was stored at −80 °C until further use.

#### cDNA synthesis

To screen for RNA viruses, RNA concentration and purity were determined using a NanoDrop ND-1000 spectrophotometer. The cDNA was synthesized using 500 ng of RNA and random hexamers and SuperScript II Reverse Transcriptase (Life Technologies; Merelbeke, Belgium) according to the manufacturer’s instructions. The cDNA was stored at 20 °C, until further use.

#### Parasite screening and molecular identification

PCR with broad range primers (if possible) was used to detect a wide variety of parasites: Protozoan parasites are screened with Neogregarine primers to detect *A. bombi* and Leishmaniinae primers to detect *Crithidia* spp. and *Lotmaria passim* (technical details are described in Meeus *et al*.^40^); microsporidia was screened with the universal primers of Fernández *et al*.^41^. For *Nosema* species identification, we designed external primers for nested PCR (Supplementary Table 1) and performed Sanger sequencing on the amplicon obtained after PCR with the internal primers of Weiss & Vossbrinck^42^. The Fungus *Ascosphaera* spp. was screened with genus-broad primers described by James & Skinner^43^ and the bacteria *P. larvae* with specific primers of Dobbelaere *et al*.^44^. Positive and negative controls were added in all PCR reactions except for *P. larvae*. The viruses IAPV, DWV, KBV and ABPV were checked by multiplex PCR as described in Meeus *et al*.^45^ and confirmed by simplex virus-specific primers as described in Sguazza *et al*.^46^. SBV, CBPV and BQCV were checked by simplex virus-specific primers as described in Sguazza *et al*.^46^.

PCR products were visualized on a 1% agarose gel by staining it in ethidium bromide and submitted to UV illumination for checking the DNA amplification. All positive pathogens retrieved on agarose gel were sent for sequencing (by LGC genomics, Berlin, Germany) in the forward direction to confirm the species. The BlastN against the nucleotide collection was performed to check the percentage match for all sequences. For known honey bee and bumble bee parasites we provide the species names, while for the others, we provide the tentative species names and the Genbank accession number of the matching sequence. The Leishmaniinae positive had very faint bands and the quality of the electropherograms was not high enough to guarantee optimal species differentiation. For primers and PCR conditions (see Supplementary Table 1).

### Infection experiments

#### Experimental design

Seeing the high diversity of parasites found in the honey bee-collected pollen and knowing that often high viral particle dosages are needed to establish oral infection^6,47^, we decided to focus on the study of the infection potential of parasites. In order to check for the infection potential of parasites within honey bee-collected pollen, we used a subset of pollen batches used for the prevalence study described above. Batches containing various parasites were selected to increase parasite repeats within the common microcolonies, when possible. All parasites present in the pollen were used in the infection experiment. A pollen ball (approximately 30 g) was provided to micro-colonies (i.e. small colony of 5 newly emerged workers of which one female will become dominant and start laying eggs). Bumble bees were provided by the company Biobest (Westerlo, Belgium). In total, we tested 8 micro-colonies and 7 parasites. We had one micro-colony control with 15 kGy-radiated pollen; the other micro-colonies received pollen naturally contaminated with at least one of the following parasites *A. bombi* (n = 4 colonies), *Crithidia* spp. (n = 5), *N. ceranae* (n = 2), *N. thomsoni* (n = 1), *Microsporidium* sp. *Oise* (n = 2), *P. larvae* (n = 1) and *A. apis* (n = 4). Pollen balls were made by mixing pollen pellets with sugar syrup at 40% (w/v). All micro-colonies were kept with sugar syrup (Biogluc) *ad libitum* under standardized laboratory conditions at 30 °C and continuous darkness^48^.

#### Infection status of bumble bees feeding on parasite-contaminated pollen

The infection status of 2 adults and 3 larvae (4^th^ stage) per colony was determined on fifteen and twenty days after colony initiation, respectively. Larvae and adults were dissected in order to obtain the gut and internal organs (mainly fat body). The sampling of body parts was to ensure that we could identify true infections for those parasites infecting inside the hemocoel. Each individual tissue was then put separately in 2 ml-Eppendorf tube with 800 µl of RLT buffer supplemented with β-mercapto ethanol (100/1; v/v) (RNeasy Mini Kit; Qiagen) and stored at −80 °C. Before extraction, samples were thawed in an incubator at 37 °C for 10 min with shaking (300 rpm). After incubation, samples were centrifuged during 2 min at 2000 g. The samples were homogenized for 2 min at 300 rpm and for 2 min at 200 rpm with 0.1 mm-diameter zirconia beads and two 5 mm-diameter metal beads in a Qiagen TissueLyser. The mixture was centrifuged for 2 min at 20000 g. DNA extraction on 200 µl of the supernatant and PCRs are performed as described previously for the pollen above.

#### Infectivity of *Ascosphaera apis* in bumble bee larvae

In order to check if *A. apis* was replicating in larvae, we checked them by microscopy and qPCR.

#### Microscopic identification and morphological measurements

Three larvae potentially manifesting symptoms from sealed cocoons were screened for vegetative or reproductive stages of *A. apis*. Each larva was sampled from different micro-colonies fed with pollen contaminated with *A. apis*. The larvae exhibiting symptoms were slide mounted for microscopy in distilled water using Wild Heerbrugg Switzerland M20 microscope. Pictures were taken by using Leica DFC295 microscope camera and Leica Application Suite version 3. Measurements of the parasite’s morphology were performed using ImageJ 1.48 software (US National Institutes of Health, http://imagej.nih.gov/ij/download.html).

#### Molecular identification and phylogenetic analysis

Symptomatic larvae were submitted to DNA extraction as described before using (RNeasy Mini Kit; Qiagen). PCR was performed by using the primers as described by James & Skinner^43^. PCR products were submitted to a 1% agarose gel and stained with ethidium bromide for checking the DNA amplification and intensification. All ITS sequences of *Ascosphaera* fungi found in dead larvae presenting symptoms, as well as from honey bee-collected pollen and living larvae samples were BLAST against the database of the website of the National Center for Biotechnology Information (NCBI) (https://blast.ncbi.nlm.nih.gov/Blast.cgi) to confirm species identity. Sequence data were checked for errors, manually edited and compared to reference sequence of *A. apis* from the American Type Culture Collection. These sequences including different *Ascosphaera* species as deposited in Genbank database, were aligned (By Muscle) and subjected to a phylogenetic analysis processed with MEGA 7 software.

#### Pathogen quantification

The quantification of *A. apis* was checked by qPCR analysis among three different sources: all the contaminated pollen batch samples with the fungus (n = 6), infected living larvae (n = 9) and symptomatic dead larvae (n = 3). *Ascosphaera apis* quantification was performed by using a CFX96 Real-Time PCR Detection system (Bio-Rad, Hercules, CA, USA), performing each reaction in triplicate. The total reaction volume of 20 µL contained 10 µL of GoTaqr qPCR Master Mix, (Promega, Madison, WI, USA), 1 µL (10 µM) Forward and 1 µL (10 µM) Reverse primer targeting the 18S rRNA gene (see Supplementary Table 1) and 8 µL of diluted DNA. Nuclease free water was used as a negative control (NTC). The standard curve was obtained from the highest infected sampled and was serially diluted (E = 93.2%; R^2^ = 0.995).

### Statistical analysis

The pathogen prevalence was determined per locality (country) and plotted using the software SigmaPlot 13.0. The difference in pathogen load among pollen, living larvae and dead larvae exhibiting symptoms was subjected to an Independent samples *t*-test (*P* < 0.05), using the software SPSS v.25 and graphically designed on SigmaPlot 13.0.

## Supplementary information

Supplementary table 1

## Data Availability

The datasets generated and analyzed in this study are available from the corresponding author on reasonable request.
