# Supplementary material for: Honey bee-collected pollen is a potential source of Ascosphaera apis infection in managed bumble bees
Source: Sci Rep. 2019 Mar 12;9:4241. doi: 10.1038/s41598-019-40804-2 (PMC6414677; doi:10.1038/s41598-019-40804-2)
Supplement: Supplementary file 1 — Supplementary table 1 [file 41598_2019_40804_MOESM1_ESM.pdf]

Supplementary information

Honey bee-collected pollen is a potential source of *Ascosphaera apis* infection in managed bumble bees

Kleber de Sousa Pereira, Ivan Meeus, Guy Smagghe

Ghent University, Faculty of Bioscience Engineering, Department of Plants and Crops, Lab of Agrozoology, Coupure Links 653, Ghent B-9000, Belgium.

Corresponding author E-mail: kleber.ds.pereira@gmail.com; ivan.meeus@UGent.be; guy.smagghe@UGent.be

Supplementary information

Supplementary table 1. List of PCR, qPCR primers and reaction conditions.

Target	Target gene	Primer name	Primer sequence (5'-3')	Product size (bp)	Temperature profile	Refs.*
Crithidia spp.	18S rRNA	SEF<br>SER	CTTTTGGTCGGTGGAGTGAT<br>GGACGTAATCGGCACAGTTT	417-20	94°C 2:00; 35x(94°C 0:30, 57°C 0:30, 72°C 0:45); 72°C 3:00	1
Apicystis bombi	18S rRNA	NeoF<br>NeoR	CCAGCATGGAATAACATGTAAGG<br>GACAGCTTCCAATCTCTAGTCG	260	94°C 2:00; 35x(94°C 0:30, 57°C 0:30, 72°C 0:45); 72°C 3:00	1
Apidae control	18S rRNA	*ApidaeF*<br>*ApidaeR*	AGATGGGGGCATTCGTATTG<br>ATCTGATCGCCTTCGAACCT	130	94°C 2:00; 35x(94°C 0:30, 57°C 0:30, 72°C 0:45); 72°C 3:00	1
Nosema spp.	16S rRNA	Nos-For<br>Nos-Rev	TATGCCGACGATGTGATATG<br>CACAGCATCCATTGAAAACG	250	94°C 2:00; 35x(94°C 0:30, 60°C 0:30, 72°C 0:30); 72°C 4:00	2
Nosema spp.	ssrRNA	SSU18SF<br>SS1537R	GTTGATTCTGCCTGACGT<br>TTATGATCCTGCTAATGGTTC	1350	94°C 5:00; 35x(94°C 1:00, 50.7°C 1:00, 72°C 1:00); 72°C 10:00	3
Nosema spp.<br>*Nested PCR*	ssrRNA	SSU18SNestF<br>SS1537NestR	ACCCATGCATGTTTTTGAAG<br>CAAAGAACAGGGACACATTCA	115	94°C 5:00; 35x(94°C 1:00, 50.7°C 1:00, 72°C 1:00); 72°C 10:00	This study
***Ascosphaera* spp.**	5.8SrDNA	AscoAll1<br>AscoAll2	GCACTCCCACCCTTGTCTA<br>GAWCACGACGCCGTCACT	450	94°C 10:00; 30X(94°C 0:45, 62°C 0:45, 72°C 1:00); 72°C 5:00	4
Paenibacillus larvae	16SrRNA	PaenibFor<br>PaenibRev	CTTGTGTTTCTTTCGGGAGACGCCA<br>TCTTAGAGTGCCCACCTCTGCG	110	95°C 2:00; 30x(93°C 1:00, 55°C 0:30, 72°C 1:00); 70°C 5:00	5
ABPV	DTLKD	MAF<br>MAR	ATGTGGGTGGATACATTAAAGGA<br>TTGTTGCCGGATTACCAGA	500	94°C 2:00; 35x(94°C 0:30, 57°C 0:30, 72°C 0:45); 72°C 3:00	6
KBV	DTLKD	MKF<br>MKR	GTGGGTCGACACATTGAAAGA<br>TTGTTGCGGGGTTTCCTGA	450	94°C 2:00; 35x(94°C 0:30, 57°C 0:30, 72°C 0:45); 72°C 3:00	6
IAPV	DTLKD	MIF<br>MIR	AATGTGGGTTGATACATTGAAAGA<br>CGTTGCGGGATTTCCAGA	450	94°C 2:00; 35x(94°C 0:30, 57°C 0:30, 72°C 0:45); 72°C 3:00	6
DWV	DTLKD	MDF<br>MDR	ATATTCACGGATTGTTTGAAAGA<br>GACAGCTTCCAATCTCTAGTCG	607	94°C 2:00; 35x(94°C 0:30, 57°C 0:30, 72°C 0:45); 72°C 3:00	6
IAPV	Polyprotein	AIVf<br>IAPVr	GGTGCCCTATTTAGGGTGAGGA<br>GGGAGTATTGCTTTCTTGTTGTG	158	94°C 5:00; 35x(94°C 0:30, 56°C 0:30, 72°C 0:45); 72°C 10:00	7
DWV	Capsid proteins	DWVf<br>DWVr	TGGTCAATTACAAGCTACTTGG<br>TAGTTGGACCAGTAGCACTCAT	269	94°C 5:00; 35x(94°C 0:30, 56°C 0:30, 72°C 0:45); 72°C 10:00	7
SBV	Polyprotein	SBVf<br>SBVr	CGTAATTGCGGAGTGGAAAGATT<br>AGATTCCTTCGAGGGTACCTCATC	342	94°C 5:00; 35x(94°C 0:30, 56°C 0:30, 72°C 0:45); 72°C 10:00	7
ABPV	Polyprotein	AIVf<br>ABPVr	GGTGCCCTATTTAGGGTGAGGA<br>ACTACAGAAGGCAATGTCCAAGA	460	94°C 5:00; 35x(94°C 0:30, 56°C 0:30, 72°C 0:45); 72°C 10:00	7
BQCV	Polyprotein	BQCVf<br>BQCVr	CTTTATCGAGGAGGAGTTCGAGT<br>GCAATAGATAAAGTGAGCCCTCC	536	94°C 5:00; 35x(94°C 0:30, 56°C 0:30, 72°C 0:45); 72°C 10:00	7
CBPV	RNA polymerase	CBPVf<br>CBPVr	AACCTGCCTCAACACAGGCAAC<br>ACATCTCTTCTTCGGTGTCAGCC	774	94°C 5:00; 35x(94°C 0:30, 56°C 0:30, 72°C 0:45); 72°C 10:00	7

Supplementary information

Ascosphaera spp.	18SrRNA	*Asc18SqPCRF*<br>*Asc18SqPCRR*	CGATGGAAGTTTGAGGCAAT<br>CTCGGCCAAGGTGATGTACT	103	94°C 10:00; 30x(94°C 0:45, 62°C 0:48, 72°C 1:00); 72°C 5:00	This study

1. Meeus, I., de Graaf, D. C., Jans, K. & Smagghe, G. Multiplex PCR detection of slowly-evolving trypanosomatids and neogregarines in bumblebees using broad-range primers. *J. Appl. Microbiol.* **109**, 107-115 (2010).
2. Fernández, J. M., Puerta, F., Cousinou, M., Dios-Palomares, R., Campano, F. & Redondo,L. Asymptomatic presence of *Nosema* spp. in Spanish commercial apiaries. *J. Invertebr. Pathol.* **111**, 106-110 (2012).
3. Weiss, L. M. & Vossbrinck, C. R. in *The microsporidia and microsporidiosis* (Wittner M, Weiss LM, editors) 129-171 (Washington, DC, USA: American Society of Microbiology, 1999).
4. James, R. R. & Skinner, J. S. PCR diagnostic methods for *Ascosphaera* infections in bees. *J. Invertebr. Pathol.* **90**, 98-103 (2005).
5. Dobbelaere, W., de Graaf, D. C. & Peeters, J. E. Development of a fast and reliable diagnostic method for American foulbrood disease (*Paenibacillus larvae* subsp. larvae) using a 16S rRNA gene based PCR. *Apidologie* **32**, 363-370 (2001).
6. Meeus, I., Smagghe, G., Siede, R., Jans, K. & de Graaf, D. C. Multiplex RT-PCR with broad-range primers and an exogenous internal amplification control for the detection of honeybee viruses in bumblebees. *J. Invertebr. Pathol.* **105**, 200-203 (2010).
7. Sguazza, G. H., Reynaldi, F. J., Galosi, C. M. & Pecoraro, M. R. Simultaneous detection of bee viruses by multiplex PCR. *J. Virol. Methods* **194**, 102-106 (2013).
